# Prevalence and correlates of intimate partner violence, before and during pregnancy among attendees of maternal and child health services, Enugu, Nigeria: mixed method approach, January 2015

**DOI:** 10.11604/pamj.supp.2019.32.1.13287

**Published:** 2019-01-25

**Authors:** Charity Chinyere Ezeudu, Onoja Akpa, Ndadilnasiya Endie Waziri, Abisola Oladimeji, Elizabeth Adedire, Ibrahim Saude, Patrick Nguku, Peter Nsubuga, Olufunmilayo Ibitola Fawole

**Affiliations:** 1 Nigeria Field Epidemiology and Laboratory Training Program, Abuja, Nigeria; 2 Enugu State Ministry of Health, Enugu, Nigeria; 3 University of Ibadan, Ibadan, Nigeria

**Keywords:** Intimate partner violence, prevalence, complications, women, injuries, infidelity, poverty, controlling behaviour, pregnancy, health services

## Abstract

**Introduction:**

Intimate Partner Violence (IPV) is an under-reported public health problem. This study determined the prevalence of IPV and types of IPV, complications and factors associated with IPV among women accessing health services.

**Methods:**

we conducted a cross-sectional survey of 702 women accessing maternal and child health services in Enugu State, Nigeria using multi-stage sampling technique. Quantitative data was collected using semi-structured questionnaire, qualitative data by key informant interview (KII). We analysed data using descriptive statistics, bivariate and multivariate logistic regression analysis. The level of statistical significance was set at p-value < 0.05. Qualitative data was analysed using thematic content analysis.

**Results:**

mean age of respondents was 27.71 ± 5.14 years and 654 (93.2%) were married. Prevalence of IPV, a year before last pregnancy, was 307 (43.7%) and during last pregnancy was 261 (37.2%). Frequent involvement in physical fights with other men, controlling behaviour and younger aged partners (< 40 years) were independent predictors of IPV experience both before and during pregnancy. Independent predictors of IPV experience before and during pregnancy were younger aged partners (< 40 years). [Adjusted Odds Ratio AOR 1.72; 95% confidence interval (CI) = 1.17, 2.53], partner having controlling behaviour AOR 2.24; 95% C.I=1.51-3.32) and Partner's frequent involvement in physical fights (AOR 2.29; 95% C.I = 1.43-3.66). Having a male child and married/cohabiting were protective against violence. KII revealed poverty, lack of education and infidelity as common triggers of IPV.

**Conclusion:**

the prevalence of IPV and types of IPV was high and the predisposing factors of IPV in Enugu were multifactorial. Couple counselling sessions that focus on non-violence conflict resolution techniques is crucial to end IPV.

## Introduction

The term "intimate partner violence" (IPV) describes physical violence, sexual violence, stalking and psychological aggression (including coercive acts) by a current or former intimate partner [1]. An intimate partner is a person with whom one has a close personal relationship. Examples of intimate partners include current or former spouse, boyfriends or girlfriends, dating partners, or sexual partners [1]. Violence against women is a global concern and it is estimated that one in every five women will experience some form of violence in their lifetime [2]. A report by the London School of Hygiene and Tropical Medicine documented that 35% of women worldwide have experienced physical and sexual intimate partner violence (IPV) [3]. The 2013 Nigeria Demographic and Health Survey (DHS) revealed that 25% of women had experienced emotional, physical, or sexual violence from their spouse and 19% had experienced one or more of these forms of violence in the past 12 months [4]. Based on the DHS report, women residing in Enugu State had the highest proportion (47.4%) of emotional intimate partner violence among the women in the South East zone and the fourth highest among the 36 states of Nigeria [4].

Violence against women hinders development and has intergenerational consequences. Women and girls constitute one half of the human capital available to reduce poverty and achieve development, but gender-based violence undermines women's education and empowerment opportunities. It also affects the development prospects of families and communities which are fundamental to achieving the Sustainable Development Goals [5]. Several studies have shown rural urban variation in the prevalence of IPV among women. A comparative, cross sectional study of women residing in urban and rural communities in Enugu State found high prevalence of domestic violence among rural women than urban women (97% versus 81, P < 0.001) [6]. This study also found that the high prevalence of IPV among rural women than urban women was because rural women were less likely to be educated, less likely to be employed, have more polygamous marital relationship and higher parity [6]. Some of the most consistent risk factors associated with IPV in available literatures are young age, male dominance in the family, man having multiple partners, personality disorder, past history of abusing partner, poverty and low self-esteem. There is dearth of knowledge on perpetrators characteristics that may be associated with violence [7].

Since most women do not report the experience of abuse, identifying risk factors for IPV is an important public health measure to reduce the morbidity and mortality associated with IPV [8]. Identifying the different forms of violence experienced by the women at the different times of their reproductive life cycle is crucial to obtain a good understanding of violence experienced by women and identify appropariate prevention measures. This study build upon previous research in that experience of abuse pre-pregnancy and during pregnancy are separated to guide appropriate prevention strategies [7]. There is dearth of knowledge on the characteristics of the perpetrators of violence, so this study describes perpetrators characteristics to predict partners that are likely to be violent. There is need to conduct research on the causes of violence against women in different cultures and in different circumstances. This study provides useful baseline information for implementing intervention programmes aimed at preventing violence and its complications. The study identified the prevalence and risk factors for IPV before and during pregnancy and associated complications among mothers attending maternal and child health services in Enugu, Nigeria.

## Methods

**Study area:** the study was conducted in Enugu State, in the South Eastern region of Nigeria. Economically, the state is predominantly rural with a large proportion of the working population being farmers. However in the urban areas trading is the dominant occupation. The state is made up of three Senatorial Zones, seven health districts and 17 Local Government Areas.

**Study design:** we conducted a health facility based on a cross-sectional study from January to December 2015 to determine the prevalence and correlates of IPV before and during pregnancy among attendees of maternal and child health services, Enugu State.

**Study population:** the study population were women attending postnatal or child immunization clinics for care. The eligibility criteria were mothers from those who had just delivered to three months postpartum.

**Sample size:** using the formula for calculating sample size of a cross sectional survey assuming a prevalence of IPV against women during pregnancy in Edo state, Nigeria of 28.3% [9]; confidence level of 95%; power of 80%; precision of 0.05, sample size of 636 was calculated which was increased to 700 to adjust for 10% non-response rate, however 702 questionnaires were completely filled and returned.

**Sampling technique:** we employed a four-staged sampling technique for collection of the quantitative data. We randomly selected by balloting one of the three senatorial zones in the state. The selected senatorial zone has three health districts that are covering 5 local government areas (LGAs). There are 20 secondary health facilities and 136 primary health facilities distributed within the five LGAs. Two health districts were randomly selected by balloting from the selected senatorial zone. Two health facilities were purposively selected from each LGA, to make a total of 10 health facilities. The number of women to be interviewed was proportionately allocated to the health facilities. The total number of women registered in each health facility were divided by the total number of women in all the 10 health facilities that was sampled and this fraction was multiplied by the sample size.. All consecutive women accessing the immunization and post-natal services were interviewed in each health facility until the desired sample size was reached. For the qualitative method, we adopted a purposive sampling technique and selected four non-governmental organizations that manage cases of intimate partner violence.

**Data collection:** we used a mixed-method for data collection. An 86-item interviewer administered semi-structured questionnaire was used to collect quantitative data. Six trained research assistants and the investigators collected data. The questionnaire collected data on respondents and their partners' socio-demographic characteristics, respondents' experience of IPV, types of IPV experienced, health problems arising from IPV and potential risk factors associated with IPV before and during pregnancy. Data was collected between June to September 2015. Qualitative data were collected by the principal investigator and two research assistants using a key informant guide, and the respondents were two key representatives (project coordinators, legal advisers, field coordinators, chairpersons and project assistants ) each of five non-governmental in Enugu State that deal on issues of domestic violence KII interviews were conducted to better understand and explore in greater depth, the triggers of IPV, coping strategies of the victims, complications arising from IPV and suggested solutions to end violence.

**Operational definitions:** IPV was defined as physical, sexual, psychological or economic abuse by a current or former partner or spouse. It includes both spouses and dating partners, in current and former relationships [10]. Physical violence was defined as the intentional use of physical force with the potential for causing death, disability, injury or harm. The physical violence included, but not limited to scratching, pushing, shoving, throwing, grabbing, biting, choking, shaking, slapping, punching, burning, use of a weapon and use of restraint or one's body, size, or strength against another [11]. Sexual violence was defined as the use of physical force to compel a person to engage in a sexual act against his or her will, whether or not the act is completed. It is an attempted or completed sex act involving a person who is unable to understand the nature or condition of the act, to decline participation, or to communicate unwillingness to engage in the sexual act, e.g., because of illness or disability [11].

Psychological also called emotional violence was defined as trauma to the victim caused by acts, threats of the act, or coercive tactics. Psychological violence can include, but is not limited to, humiliating the victim, controlling what the victim can and cannot do, withholding information from the victim, deliberating doing something to make the victim feel diminished or embarrassed, isolating the victim from friends and family [11]. Economic violence was defined as denying the victim access to money or other basic resources, controlling the victim's finances to prevent them from accessing resources, working or maintaining control of earnings, achieving self-sufficiency and gaining financial independence [12]. Controlling behavior was defined as trying to keep her from seeing friends or family, got angry if he speaks with another man, suspects her of unfaithfulness or monitors her movement. Perceived negative attitude of the mother-in-law was defined as involvement in decision making that may be detrimental to the respondents. Frequent physical fighting was defined as having been involved in fights with other men of ≥ 12 times per year.

**Study variables:** the main outcome variable was any experience of IPV 12 months before pregnancy and also during the current pregnancy. This included physical, sexual, psychological or economic violence. Independent variables were socio-demographic characteristics of the respondents and partner: age, religion, marital status, family type (polygamous and monogamous), educational level and occupation.

**Data analysis:** we reviewed all completed questionnaires before electronic data entry. We conducted univariate analysis to obtain frequencies and proportions, bivariate and multivariate logistic regression analysis to identify associations and independent predictors of IPV before and during pregnancy. P value set at < 0.05. Data analysis was performed using SPSS version 20. Qualitative data from key informant interview were thematically analyzed.

**Ethical considerations:** we obtained ethical clearance for the study from Enugu State Ministry of Health Ethical Review Board with Ref Number -MH/MSD/EC/0173. Written informed consent was obtained from each respondent. We also obtained verbal and written informed consent from an adult related to mothers below 15 years of age. Confidentiality of the respondents was assured and maintained during and after the study.

## Results

### Quantitative

Socio demographic characteristics: mean age of the respondents was 27.71 ± 5.14 years and the age group 25-29 has the most respondents (n/N, 40.5%). A total of 233 (33.2%) respondents were unemployed. Nearly all 684 (97.7%) of the study participants were Christians while 654 (93.2%) of the women were married (Table 1).

**Table 1:** socio-demographic characteristics of respondents interviewed on the predictors of intimate partner violence in Enugu State, Nigeria, December 2015

Variable		
Age distribution (in years)	Number of respondents (N=702)	Proportion (%)
15 - 19	21	3.0
20 - 24	170	24.2
25 - 29	284	40.5
30 - 34	152	21.7
35 - 39	55	7.8
40 - 44	16	2.3
45 - 49		
Religion	4	0.5
Christianity	684	97.4
Islam	6	0.9
Traditional	7	1.0
Others		
Marital status	5	0.7
Single	42	6.0
Married/cohabiting	655	93.3
Divorced/separated	5	0.7
Educational level		
None	12	1.7
Primary	67	9.5
Secondary	367	52.3
Post-secondary		
Occupation	256	36.5
Unemployed	233	33.2
Trader	195	27.8
Artisan (tailor, hairdressing)	181	25.8
Civil servant/government	58	8.3
Farmer	15	2.1
Others (National Youth Service Corp)	20	2.8

Prevalence of Intimate Partner Violence before and during the last pregnancy: the overall prevalence of IPV was 48.3% (339). Experience of IPV 12 months before the last pregnancy was reported by 307 (43.7%) out of 702 respondents. IPV experience during the last pregnancy was reported by 261 (37.2%) out of 702 respondents. IPV during pregnancy is lower than before pregnancy and this was statistically significant (P <0.001). The overall prevalence of physical violence was 26.2% (184), sexual violence 27.8% (195), psychological violence 36.9% (259) and economic violence 23.9% (168). One hundred and fifty-five (50.5%) respondents had experienced physical violence in the 12 months before pregnancy while 42.5% (111) respondents experienced physical violence during pregnancy. A larger proportion of the respondents had experienced psychological (emotional) violence before pregnancy, (67.4% (207) and during pregnancy 65.9% (172).

### Health problems arising from IPV

**Figure 1:**
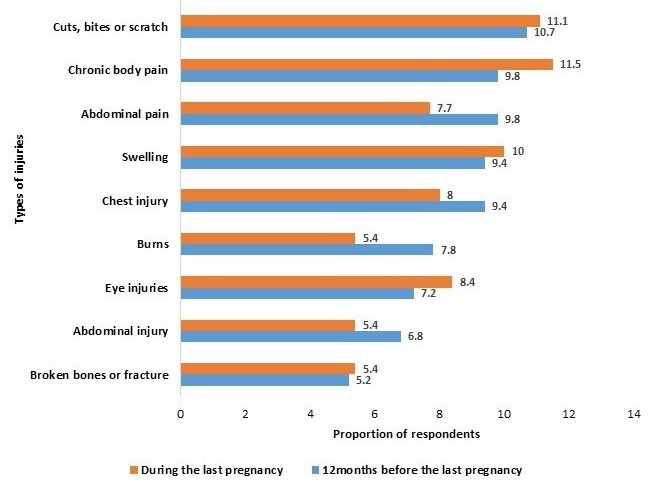
physical injuries sustained by victims of intimate partner violence 12 before and during pregnancy in Enugu State, December, 2015

A large proportion (n = 307, 43.7%) of the victims of IPV also reported physical injuries such as fractures 5.2% (16), chest injuries 9.4% (29), burns 7.8% (24) and abdominal injuries 6.8% (21) 12 months before the last pregnancy. Those that experienced IPV during the last pregnancy (261) reported abdominal injuries 5.4% (14), burns 5.4% (14, eye injuries 8.4% (22), fractures 5.4% (14) and chest injuries 8.0% (21) (Figure 1).

### Factors associated with IPV experience

Factors that were significantly associated with IPV on bivariate analysis before pregnancy were partner's history of controlling behaviours (Crude Odds Ratio (COR) = 2.42, Confidence Interval (CI), 1.65, 3.52), history of frequent physical fights (COR = 2.67, CI: 1.69, 4.20) and younger aged partners (< 40 years) (COR = 1.63, CI: 1.13, 2.34 (Table 2). During pregnancy, partner's history of controlling behaviours (COR = 3.09, CI: 2.11, 4.52) history of frequent physical fights (COR = 2.69, CI: 1.72, 4.19) and younger aged partners (< 40 years) (COR = 1.64, CI: 1.12, 2.40) were also significantly associated with IPV (Table 3).

**Table 2:** bivariate analysis of factors associated with intimate partner violence before among attendees of maternal and child health services in Enugu State, Nigeria, December 2015

History of violence before pregnancy				
	Experienced violence N = 307 N (%)	Did not experience violence N = 395 N (%)	COR (95%)	P value
**Marital status**				
Single or divorced	23 (48.9)	24 (51.1)	1.25 (0.70, 2.26)	0.456
Married or cohabiting	284 (43.4)	371 (56.6)	1	
**Occupation**				
Unemployed	6 (35.3)	11 (64.7)	0.70 (0.25, 1.90)	0.477
Employed	301 (43.7)	384 (56.3)	1	
**Have a male child**				
Yes	221 (46.9)	250 (53.1)	1.49 (1.08, 2.06)	0.015
No	86 (37.2)	145 (62.8)	1	
**Partner’s infidelity**				
Yes	20 (57.1)	15 (42.9)	2.18 (1.08, 4.38)	0.026
No	156 (38.0)	255 (62.0)	1	
**Perceived negative attitude of the mother in law**				
Yes	84 (52.8)	75 (47.2)	1.61 (1.13, 2.29)	0.009
No	223 (41.1)	320 (58.9)	1	
**Controlling behaviour**				
Yes	85 (61.2)	54 (38.8)	2.42 (1.65, 3.54)	<0.001
No	222 (39.4)	341 (60.6)	1	
**Previous history of frequent physical fights with other men**				
Yes	60 (64.5)	33 (35.5)	2.67 (1.69, 4.20)	<0.001
No	247 (40.6)	362 (59.4)	1	
**Partner’s age**				
< 40	250 (46.5)	288 (53.5)	1.63 (1.13, 2.34)	0.008
≥40	57 (34.8)	107 (65.2)	1	
**History of violence during pregnancy**				
**Marital status**	27 (57.4)	20 (42.6)		
Single or divorced	234 (35.7)	421 (64.3)	2.43 (1.33, 4.43)	0.003
Married or cohabiting				
**Occupation**	8 (47.1)	9 (52.9)	1	
Unemployed	253 (36.9)	432 (63.1)	1.52 (0.58, 3.98)	0.394
Employed				
**Have a male child**	179 (38.0)	292 (62.0)	1	
Yes	82 (35.5)	149 (64.5)	1.11 (0.80, 1.55)	0.519
No				
**Partner’s infidelity**	20 (57.1)	15 (42.9)	1	
Yes	135 (32.8)	276 (67.2)	2.73 (1.35, 5.49)	0.003
No				
**Perceived negative attitude of the mother in law**	73 (45.9)	86 (51.4)	1	
Yes	188 (34.6)	355 (65.4)	1.60 (1.12, 2.29)	0.009
No				
**Controlling behavior**	82 (59.0)	57 (41.0)	1	

**Table 3:** bivariate analysis of factors associated with intimate partner violence during pregnancy among attendees of maternal and child health services in Enugu State, Nigeria, December 2015

History of violence during pregnancy				
	Experienced violence N = 307		Did not experience violence N = 395	
	N (%)	N (%)	COR (95%)	P value
**Marital status**				
Single or divorced	27 (57.4)	20 (42.6)	2.43 (1.33, 4.43)	0.003
Married or cohabiting	234 (35.7)	421 (64.3)		
**Occupation**			1	
Unemployed	8 (47.1)	9 (52.9)	1.52 (0.58, 3.98)	0.394
Employed	253 (36.9)	432 (63.1)	1	
**Have a male child**				
Yes	179 (38.0)	292 (62.0)	1.11 (0.80, 1.55)	0.519
No	82 (35.5)	149 (64.5)	1	
**Partner’s infidelity**				
Yes	20 (57.1)	15 (42.9)	2.73 (1.35, 5.49)	0.003
No	135 (32.8)	276 (67.2)	1	
**Perceived negative attitude of the mother in law**				
Yes	73 (45.9)	86 (51.4)	1.60 (1.12, 2.29)	0.009
No	188 (34.6)	355 (65.4)	1	
**Controlling behavior**				
Yes	82 (59.0)	57 (41.0)	3.09 (2.11, 4.52)	< 0.001
No	179 (31.8)	384 (68.2)	1	
**Previous history of frequent physical fights with other men**				
Yes	54 (58.1)	39 (41.9)	2.69 (1.72, 4.19)	< 0.001
No	207 (34.0)	402 (66.0)		
**Partner’s age**			1	
< 40	214 (39.8)	324 (60.2)	1.64 (1.12, 2.40)	0.008
≥ 40	47 (28.7)	117 (71.3)	1	

### Independent predictors of IPV experience before and during pregnancy

In the multivariate logistic regression analysis, independent predictors of IPV before pregnancy, were controlling behaviour (AOR = 2.24, 95% CI: 1.51, 3.32), history of frequent physical fights (AOR = 2.29, CI: 1.43, 3.66) and partners less than 40 years, [Adjusted Odds Ratio (AOR) = 1.72, 95% CI: I.17, 2.53] (Table 4). Similarly, during pregnancy, independent predictors of IPV were controlling behaviour (AOR = 2.92, CI: 1.96, 4.34), history of frequent physical fights AOR = 2.34, CI: 1.47, 3.72) and partners less than 40 years (AOR = 1.68, CI: 1.12, 2.53). Respondents with at least one male child AOR = 0.66, CI: 0.47, 0.93) and those that were married/cohabiting AOR = 0.46, CI: 0.24, 0.90) were found to be less likely to experience IPV.

**Table 4:** multivariate logistic regression model to identify independent predictors of violence before and during the last pregnancy in Enugu State, Nigeria, December 2015

^+^n = 441	Factors associated with IPV before pregnancy		Factors associated with IPV during pregnancy	
	AOR (95% CI)	P-value	AOR(95% CI)	P-value
**History of frequent physical fight with other men**				
Yes	2.29 (1.43, 3.66)	0.001	2.37 ( 1.47, 3.72)	< 0.001
No	1		1	
**Controlling behaviour**				
Yes	2.24 (1.51, 3.32)	< 0.001	2.92 (1.97, 4.34)	< 0.001
No	1		1	
**^+^Partner’s infidelity**				
Yes	1.63 (0.79, 3.39)	0.19	1.91 (0.91, 4.02)	0.09
No	1		1	
**Have a male child**				
Yes	0.66 (0.47, 0.93)	0.02	0.92 (0.64, 1.30)	0.61
No	1			
**Partner’s age**				
Yes	1.72 (1.17, 2.53)	0.01	1.69 (1.12, 2.53)	0.01
No	1			
**Marital Status**				
Married/cohabiting	0.85 (0.44, 1.63)	0.614	0.46 (0,24, 0.90)	0.02
Single/Divorced	1			
**Perceived negative attitude of the mother in law**				
Yes	1.34 (0.96, 2.01)	0.083	1.35 (0.92, 1.97)	0.13
No	1			

### Qualitative results

During the key informant interviews, we identified the following thematic areas.

**Tools used in IPV as reported by the informants:** we found out that most of the victims reported that their partner had beaten them with a horse whip, iron cable, twisted electric wire or belt buckle. Quote: *“I seized the horsewhip a man used to beat the wife, look at it, they also use broom on their wives.”*…..40 years old legal adviser.

**Triggers of IPV:** the major triggers of IPV stated by the informants were a lack of education by the partner, patriarchal nature of the society, controlling behaviour exhibited of partners, sex denial, extreme anger by partner, poverty, alcohol, nagging, infidelity, lack of sexual attraction for the wife and ignorance. Quote: *“This men beats their wife, when they are drunk” “Most of our clients are not educated”*…..30 years project assistant. *“Poverty rate is high among women in Enugu State, they are financially dependent on their partners”……*.40 years legal adviser.

**Complications of IPV:** it was reported by the NGOs that the victims presents with complications such as swollen face, buttocks and eyes, broken teeth, bleeding and unwanted pregnancy. Quote: *“Many women have come to the NGOs office with an abdominal injury because the men used the rod on the abdomen, red eye, facial bruises, vaginal bleeding due to marital rape, cuts, marks, swelling, trauma to the womb.”*…..35 years old field coordinator.

Coping strategies used by the victims: the most common coping strategies used by the victims were running away from the abuser or accepting it as a way of life. Quote: *“It is a taboo to discuss the abuse and other women will also condemn her for opening up”*….58 years old project coordinator.

Ways of reducing or preventing violence in the State: the key informants suggested violence preventive strategies such as creating of family courts in the state to settle family disputes, passage of the Gender and Equal Opportunity bill in the state, women empowerment, enacting laws prohibiting violence against women and sensitization of the community to create awareness. Quote: *“To stop violence, the Gender and Equal Opportunities bill to prevent all forms of domestic violence against women should be passed into lawin Enugu State. The government should create family courts in the state and translate existing laws and the rights of women to local languages. They should also create awareness, empowerments and education.”* …..45 years old legal adviser (Table 5).

**Table 5:** summary findings from key informant interviews conducted with officials of notable NGOs that deal with issues of violence in Enugu State, December, 2015

Thematic Areas	Respondents (Officials of Non-Governmental Organization that deal with issues of domestic violence)
Types of violence	Physical violence: The men use horse wipe belts, buckle and broom on their wives; a women with eight months old pregnancy beaten to a state of unconsciousness hair pulling, slapping, hitting her head against the wall.
	Psychological violence: The men destroy their items of trade; prevents their pregnant wives from receiving antenatal care
	Sexual violence: Marital rape and forced abortion
	Economic violence: The men forcefully collect money from their wives; the men destroy their items of trade; they refuse to give their wives money for food; they refuse to allow her take a job; refuse to pay the children’s school fees and they hide money away from their wives; the women suffer from starvation and lack of maintenance; They make unnecessary financial demands on their wives.
Triggers of violence	Lack of sexual attraction
	High poverty rate in state
	Societal norms and values
	They batter for pride
	Patrichial nature of the society
	If she refuse him sex
	Extreme anger
	Controlling behaviour
	Nagging
Complications of IPV	Swollen reddish face and eyes; Burns from hot iron and hot water; Open wounds; Vaginal tear and bleeding as a result of marital rape; Insanity; broken bones and teeth; unconsciousness; miscarriages; unwanted pregnancy
Coping strategies of the victims	The women prefers to report to their own family, husband’s family, their religious leaders and NGOs
	They also run away from the home
	Some fight back
	Staying in the marriage and accepting it as a way of life
Very few of the victims report to law enforcement agents
Solutions to end violence	Creating family courts in the state to settle family disputes
	Domesticating the violence gender inequality bill in the state
	Translating existing laws and the rights of women to local languages
	Creating employment for the men and women
	Enacting the laws on prohibiting violence against women in the state
	Sensitization to create awareness on the complications of violence on the victims, family and the society
	Organizing behavioural modification seminars for men and women.

## Discussion

This survey determined the prevalence and correlates of IPV before and during pregnancy. The overall prevalence (48.3%) of IPV was high and the prevalence both before (43.7%) and during (37.2%) pregnancy was also high. We found that controlling behaviour, history of frequent involvement in physical fights with other men and younger aged partners (< 40 years) were factors associated with IPV both before and during pregnancy in Enugu State. The women experienced the different types of IPV and as a result, they suffer from complications such as fractures, burns, vaginal tears and bleeding. The overall prevalence of IPV was high. This finding is similar to results from a cross-sectional study conducted in Southeast Nigeria, that found a high overall prevalence of IPV [13]. The prevalence of IPV and the different types of IPV was also high both before and during pregnancy, which is similar to findings from a cross-sectional study of 2042 post natal women conducted in Zimbabwe [14]. Our findings suggested that violence against women is a major public health problem in Enugu State and that pregnant women were also victimized. An explanation to this high prevalence may be because women are excluded from decision-making even within the family as a result of the strong patriarchal values of the society. Traditionally women occupy a lower position than men in the social structure, they are regarded as inferior to men and are marginalized. They are unable to speak up for their rights because of fear of threats and intimidation by the men and society [15].

Psychological (emotional) violence was the most prevalent form of violence both before and during pregnancy. This finding is consistent with a cross-sectional study of 695 women conducted in Orlu, Imo State Nigeria [16]. Psychological violence may be the predominant form of abuse during pregnancy because the partner may want to avoid physical violence which may affect the unborn child. It can also be perpatrated secretly hence less likely to draw attention of others. During pregnancy, women's psychological, nutritional and medical support from partner is important for their wellbeing and that of her baby. The findings from this study revealed that the predictors of IPV both before and during pregnancy were younger aged partners (<40 years), history of frequent involvement in physical fights with other men and controlling behaviour. Respondents with at least one son and those that were married or cohabiting were less likely to experience violence. The association between marital status and IPV is well established in the literature [7, 17]. The result of this study revealed that women that were married or co-habiting were less likely to be abused than the married during pregnancy. Male partner's controlling behaviour was found to be a strong predictor of violence both before and during pregnancy.

This finding is consistent with a WHO multi-country study that revealed that male partner's controlling behaviour was found to be a strong predictor of violence both before and during pregnancy. Violence may arise from the need to enforce power and control in a relationship and pregnancy may have a significant impact on the power dynamic of a relationship [18]. This finding is in agreement with a cross sectional population based survey conducted nationally in Nigeria with a sample size of 19,216 women which found that women whose partner exhibited controlling behaviour were more likely to be violated [19]. The partner involvement in a physical fight with other men was found to be associated with IPV both before and during pregnancy. This indicates that the partner resorts to violence to resolve conflicts in different situations. This finding is consistent with a study conducted in Harare, Zimbabwe that found that women who reported that their partners often fight with other men were more likely to be violated. We also found that younger aged partners (< 40 years) were more likely to perpetrate IPV. This finding were also in consistent with a health facility based cross-sectional study conducted in Harare Zimbabwe that also implicated frequent physical fight as a predictor for IPV [20]. The association between young men and violence may be due to lack of experience in resolving marital disputes. A peculiar finding in this study was that women with at least a male child were less likely to experience IPV before pregnancy, this shows the strong value placed on having a son. The women experienced the different types of IPV and as a result, they suffer from complications such as fractures, burns, vaginal tear and bleeding, abdominal and chest injuries.

The results of the study can be generalised to other states in the South eastern region of the country where socio cultural beliefs and women's status are similar. Our study has two limitations. First, the study was cross-sectional in design and thus, could not establish causality, but the findings are useful for planning and implementing interventions to end violence. Secondly, they may also have been recall bias because of the time lag between when the violence occured and when the interviewed was conducted. However, the questions were repeated many times and linked to events in the lives of the women to elicit accurate responses. Further research is needed on the identification of socio-cultural factors that contributes to the high prevalence of violence against women.

## Conclusion

The overall prevalence of IPV and the prevalence both before and during pregnancy was high. The risk factors were multifactorial and included controlling behaviour, frequent involvement in physical fights and younger aged partners (< 40years). The high prevalence of IPV before and during pregnancy and its association with partner's characteristics reflects the importance of couple counselling to bring about behavioural modification to end violence. Couples also need education on how to resolve conflict without resorting to violence. The majority of the victims suffered from physical health problems, hence the urgent need for the State Ministry of Health to develop a training programme for health workers on the identification and management of cases of IPV in the health facilities. There may be need to routinely screen pregnant women for abuse during antenatal visits to the health facilities. The State Government should pass laws prohibiting violence to women. There should be public enlightenment through the mass media on the negative effects of intimate partner violence against women. Partners should visit marriage counsellors for counselling on relationship training which will lead to couples expressing more gender equitable behaviour and increased communication skill.

### What is known about this topic

- Intimate Partner Violence is the commonest type of gender based violence experienced by women across the globe. Enugu state has a high prevalence of IPV to women.

### What this study adds

- The determinants of experience of IPV in pregnancy are described, as this is a time to protect the health of the mother and her unborn child;
- Provides understanding on why prevalence of IPV to women in Enugu State is high; provides information on male partner characteristics that is associated with IPV;
- Women with a son are less likely to be violated by their partner; need for couple counselling on non-violence conflict resolution strategies.

## Competing interests

The authors declare no competing interests.
